# Predictors of Acute Compartment Syndrome in Patients With Forearm Fractures: A Systematic Review

**DOI:** 10.7759/cureus.54757

**Published:** 2024-02-23

**Authors:** Ahmed AlHussain, Nouf A Almagushi, Fay Alowid, Bashayer AlObaid, Nawaf A Almagushi, Sultan N Alotaibi, Mohammad S Almosa, Mashari A Alhossan, Saif S Alanazi, Faisal Alhuwairini, Musaad M Bin Dukhi

**Affiliations:** 1 Orthopaedic Surgery, King Abdulaziz Medical City, Riyadh, SAU; 2 Department of Medicine, King Saud Bin Abdulaziz University for Health Sciences College of Medicine, Riyadh, SAU; 3 College of Medicine, King Saud Bin Abdulaziz University for Health Sciences, Riyadh, SAU; 4 Medical School, Royal College of Surgeons in Ireland, Dublin, IRL; 5 Medical School, Imam Abdulrahman Bin Faisal University, Al Khobar, SAU; 6 Orthopaedic Surgery, Security Forces Hospital, Riyadh, SAU

**Keywords:** intercompartmental pressure, fractures, forearm, acs, acute compartment syndrome

## Abstract

Acute compartment syndrome (ACS) is a critical orthopedic and traumatology emergency arising from elevated pressure within a confined osteofascial compartment, leading to compromised blood circulation and tissue ischemia. This systematic review aims to comprehensively identify and analyze the most predictable risk factors associated with ACS development in patients with forearm fractures. Published articles on ACS were meticulously searched and evaluated on reputable medical databases such as PubMed. The keywords “risk factors associated with the ACS in patients who have sustained forearm fractures”were used to create the search syntax on various databases. Data were gathered on raw prevalence, population under study, and methodology. A total of 10 articles that met the search criteria were identified and included in this review with a total of more than 300,000 patients across the studies. Fracture-related ACS was the most common, followed by soft tissue damage among patients with forearm fractures. This review underscores fractures as primary ACS catalysts, along with the role of soft tissue trauma. Meticulous consideration of these risk factors can enhance clinical decision-making, early detection, and intervention, improving patient outcomes and care quality.

## Introduction and background

Acute compartment syndrome (ACS) is one of the few orthopedics and traumatology medical emergencies that occurs when pressure builds up within a confined osteofascial compartment, exceeding perfusion pressure and causing impaired blood circulation [1]. ACS can lead to nerve and muscle ischemia and unrelenting severe pain whose cause cannot be ascertained. Medical data shows the potential of developing ischemia and, consequently, necrosis. This makes ACS a surgical emergency. The diagnosis of ACS is generally a clinical diagnosis when a patient presents with signs indicative of ACS. An intracompartmental pressure (ICP) test is performed to confirm the diagnosis of ACS. This diagnosis is considered when ICP exceeds the 30 mmHg threshold [1,2].

The most common risk factors associated with ACS include traumatic injuries, such as tibia and distal radius fractures [1], crush injuries, or severe muscle strains. Patients with open fractures in the forearm are at a higher risk of developing ACS. It can also be caused by other factors, including tight bandages or casts, excessive exercise, and certain medical conditions [1,2]. ACS is more likely to occur in specific body areas. These include the forearm, especially among athletes such as motocross racers, thigh, anterior compartment of the leg, buttocks, and shoulders. Logically, any condition that restricts the intracompartmental space and leads to excessive fluid accumulation in the muscle compartment is a risk factor for ACS. According to the National Library of Medicine (NLM) [1], special focus should be accorded to patients with a history of limb fractures because skin laceration does little to alleviate the pressure caused within the muscle fascia.

According to the NLM [1], ACS is predictable, especially among patients presenting with open Gustilo type II and III lesions in intra-articular proximal tibia fractures [3]. The condition can remain latent and painless initially. It may later manifest, leading to the absence of a distal pulse, hypoesthesia, and extreme paresis when ICP compromises arterial blood flow. This lack of oxygenated blood in the tissue induces pain, causes nerve irritation, and reduces peripheral sensation.

ACS is a surgical emergency that requires immediate attention, and, if not promptly treated, ACS can lead to serious complications, such as nerve damage, muscle death, and permanent disability [4]. Treatment often involves surgical intervention to relieve the pressure within the compartment, allowing blood flow to be restored and preventing further damage.

This systematic review aims to comprehensively identify and analyze the most predictable risk factors associated with the development of ACS in patients who have sustained forearm fractures. By systematically synthesizing existing evidence, this review aims to provide a comprehensive overview of the risk factors that contribute to the occurrence of ACS in this specific patient population. The synthesis of findings from various studies will facilitate a deeper understanding of the factors that clinicians should consider when assessing the risk of ACS following forearm fractures, enabling more accurate early identification and timely intervention.

## Review

Methodology

This systematic review was conducted and reported in line with the Synthesis Without Meta-Analysis (SWiM) in systematic reviews, a subset of the Preferred Reporting Items for Systematic Reviews and Meta-Analysis (PRISMA) reporting guidelines. The adherence to PRISMA guidelines was thoroughly examined. The checklist presented in the Appendices sheds light on the comprehensive nature of our systematic review.

Study Design

A comprehensive search strategy was developed to systematically identify relevant studies investigating the risk factors associated with the development of ACS in patients who have sustained forearm fractures. The search was conducted across three primary databases, namely, PubMed, Google Scholar, and ScienceDirect. The choice of these databases was based on their extensive coverage of medical literature and relevance to the research topic.

Data Sources and Extraction

We searched several medical databases for articles on “acute compartment syndrome” OR “compartment syndromes” that were published between 1998 and the time of this systematic review.

Keywords and a wide range of medical subject headings (MeSH) such as “forearm fracture,” “acute compartment syndrome,” or “compartment syndrome” were combined with key epidemiological terms such as survey and systematic review. Boolean words “AND” and “OR” were also utilized in the search syntax as keyword conjunctions. For further insights into the search criteria and syntax, refer to Table 1.

**Table 1:** Search criteria and syntax.

Database	Search strategy [MeSH and keywords]
PubMed	(“Forearm Injuries”[Mesh] OR “Forearm Fractures”[Mesh]) AND (“Compartment Syndromes”[Mesh] OR “Compartment Syndrome”[Title/Abstract] OR “Acute Compartment Syndrome”[Title/Abstract]) AND (“Risk Factors”[Mesh] OR “Predictive Factors”[Mesh] OR “Prognostic Factors”[Mesh])
NLM	((((((((((Forearm Injuries[MeSH Terms]) OR (Forearm Fractures[MeSH Terms]))) AND (Compartment Syndromes)) OR (Compartment Syndrome))) OR (Compartment Syndrome[Title/Abstract])) OR (Acute Compartment Syndrome[Title/Abstract])) OR (Risk Factors[MeSH Terms])) OR (Predictive Factors[MeSH Terms])) OR (Prognostic Factors[MeSH Terms])
Google Scholar	“forearm fractures” OR “forearm injuries” AND “acute compartment syndrome” AND “risk factors”
ScienceDirect	(“forearm fractures” OR “forearm injuries”) AND (“acute compartment syndrome” OR “compartment syndromes” OR “acute compartmental syndrome”) AND (“risk factors” OR “predictive factors” OR “prognostic factors”)

MeSH terms and keyword variations were used to capture relevant articles related to forearm fractures, compartment syndrome (including ACS), and risk factors. MeSH terms were employed to ensure precision in retrieving articles with specific indexing terminology.

After a thorough examination of the complete texts, a subsequent review of citations was conducted. The reference lists of all acquired articles underwent meticulous scrutiny to identify outdated research articles (i.e., published before 1998) and articles that were devoid of abstracts.

Inclusion and Exclusion Criteria

The inclusion criteria for this study encompassed articles investigating the most predictable risk factors associated with the development of ACS in patients who have sustained forearm fractures. We included full-text articles whenever feasible and applied a comprehensive eligibility criterion based on the identification of risk factors associated with ACS, either in clinical settings or through self-reported cases. The study design had to involve original quantitative data analysis.

**Figure 1:**
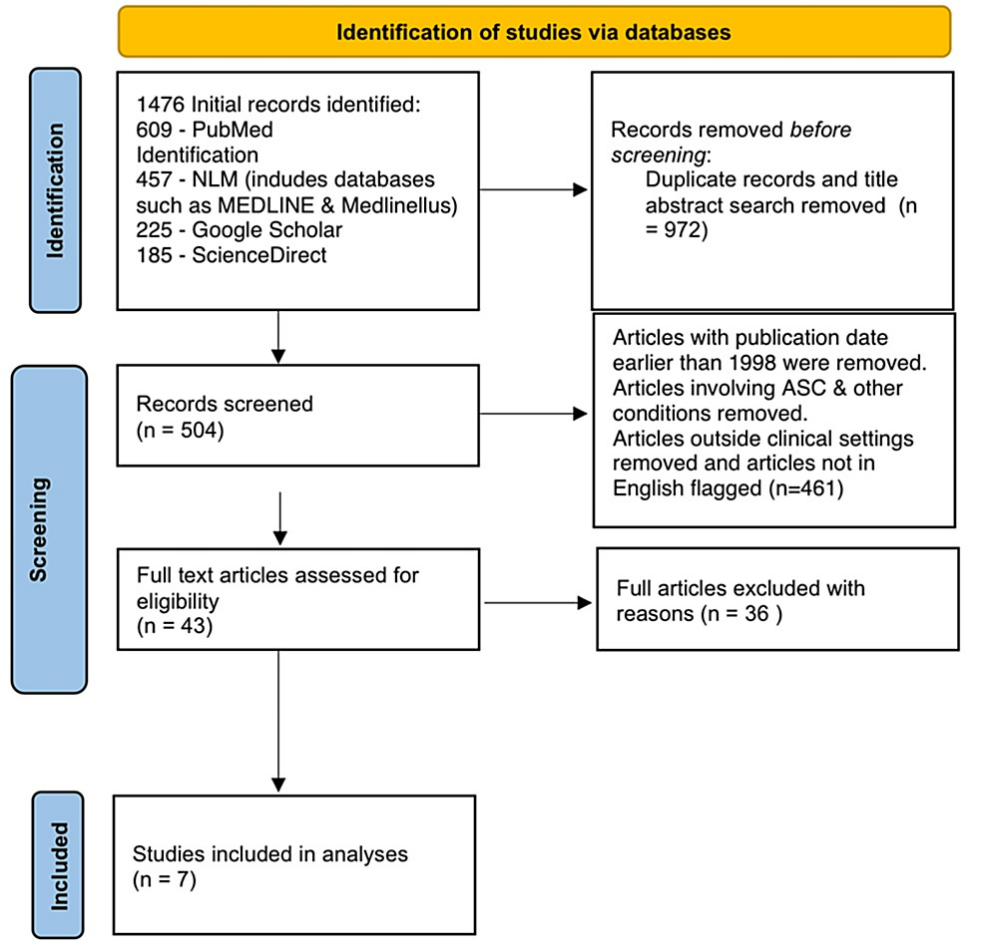
Preferred Reporting Items for Systematic Reviews and Meta-Analysis flow diagram.

Exclusion criteria included studies that combined ACS risk factor analysis with other conditions, intervention studies, and medical surveys targeting specific patient groups or disease cohorts such as diabetes. Studies were considered irrespective of their study designs. Preference was given to articles published in the English language. The screening process involved two independent researchers. The flowchart in Figure 1 outlines our study’s systematic review process, starting with 1,476 initial records and progressing through the screening stages to ultimately include seven studies in the final analysis.

Quality Assessment

An independent external evaluator employed the Epidemiological Appraisal Instrument (EAI) to assess and assign quality scores to this review. The EAI, a tool capable of evaluating the quality and methodological rigor of systematic reviews, provided a structured framework to scrutinize various facets of study design, implementation, and reporting. For this quality assessment, factors not pertinent to the aforementioned study design were excluded, resulting in a tally of 10 final articles. All identifiers, such as titles and author information, were redacted to anonymize the articles before scoring. Evaluation scoring entailed designating “Yes” (score = 2), “No” as (score = 0), and “NA” (omitted from assessment). Before scoring, a consensus was reached among all parties on the quality assessment criteria to resolve any potential disputes. Subsequently, each study underwent classification as either “High” or “Low” quality based on individual scores.

Results

The initial search of the database yielded a total of 1,476 records, of which 972 were excluded following a comprehensive evaluation of titles and abstracts against the predefined search text: “Risk factors associated with the development of acute compartment syndrome (ACS) in patients with forearm fractures.” Subsequently, a thorough assessment of the remaining 504 records led to the exclusion of 461 articles that did not meet the predetermined exclusion criteria, which encompassed studies published before 1998, non-English-language publications, and studies deemed irrelevant to the focal theme. Further full-text review indicated that 43 records aligned with the study’s search criteria, prompting a comprehensive evaluation for eligibility. Applying rigorous quality appraisal, 36 studies were further excluded, culminating in the identification and inclusion of seven studies of high quality for incorporation into this systematic review. For detailed characteristics of the selected articles, refer to Table 2.

**Table 2:** Characteristics of included articles.

N	Author	Country	Sample (n)	Age group	Risk factors
1	Yang et al., 2023 [5]	China	611	18 and above	Crush injury, level of neutrophils, and creatine kinase
2	Laverdiere et al., 2023 [6]	USA	120,556	18 and above	Open fractures, complex fractures (OTA type-C), and substance abuse disorder
4	Alshahrani et al., 2018 [7]	Saudi Arabia	77	18 and above	Renal tubular acidosis
5	McQueen and Gaston (2000) [8]	UK	164	>18 years	Fracture of the distal end of the radius in the forearm, injury to soft tissues without fracture, sex, and age
7	Shore et al., 2013 [9]	USA	212	14 years and above	Tibial shaft fractures
8	Bouklouch et al., 2022 [10]	USA	203,500	Undefined	Proximal and midshaft tibial fractures, substance abuse disorder, crush and penetrating injuries, sex, body mass index, and cirrhosis
10	Khoshhal et al., 2022 [11]	Multi-region review	684	3–75 years	History of arm fracture, soft tissue injury, and vascular injury

Fracture-Induced Acute Compartment Syndrome

All seven articles provided relevant evidence of patients’ history of fractures as the main reason for developing ACS [5,6,8,11] and identified radius, ulna, and both-bone fractures in the arm as the leading risk factors for patients who developed ACS. Overall, 50% of the articles with fracture-related ACS identified both-bone fractures as the most common cause of ACS [5,11]. Table 3 provides detailed information on the prevalence of ACS in distinct fracture types.

**Table 3:** Prevalence of acute compartment syndrome (ACS) in different fracture types.

N	Authors	Fracture type	% occurrence of ACS of the total fractured patients
1	Yang et al., 2023 [5]	Both-bone fracture	13%
2	Laverdiere et al., 2023 [6]	Open-forearm fractures	1.6%
6	Khoshhal et al., 2022 [11]	Supracondylar humerus and both-bone forearm fractures	65.4%

Soft Tissue Damage-Induced Acute Compartment Syndrome

Soft tissue trauma was identified as the second-most common cause of ACS among patients with forearm injuries in one of the seven articles reviewed [5,8]. Yang et al. [5] identified soft tissue injury as the second-most common cause of ACS after forearm structures.

Forearm fractures and soft tissue damage were therefore identified as the most common causes of ACS in the seven studies included in this systematic review. Open-arm fractures were contended as a potent cause of ACS due to conflicting logic on how the pressure would build up in an open-arm fracture. However, fractures of the tibia and diaphysis and the arm radius were identified as a common cause of ACS [5,6,8,11]. Vascular injuries and substance abuse disorders were the least common causes of ACS and were reported in only two of the seven studies included in this review [6,10].

Discussion

In the present systematic review, an assessment was conducted on the origins of traumatic compartment syndrome in the forearm, unveiling fractures as the predominant causative factor triggering forearm ACS. Ulna and radius shaft fractures emerged as notable contributors to the occurrence of ACS. Blunt soft tissue trauma exhibited elevated occurrences of forearm ACS compared to other types of soft tissue injuries. Notably, substance abuse disorders and vascular injuries exhibited the lowest propensity to induce forearm ACS.

Fractures emerged as the predominant cause of forearm ACS, highlighting the significance of their role. Among fractures contributing to ACS, fractures of the radius or ulna emerged as the most prevalent (69%), followed by supracondylar humerus and both-bone forearm fractures (65.4%) and ulna-radius fractures (58%). This underscores the notable impact of specific fracture sites on the incidence of forearm ACS. ICPs >30 mmHg were reported in 30% of the studies under review to diagnose ACS among patients presenting with a history or current forearm fractures [10,11].

We compared the results of this study with those of Khoshhal et al. who reviewed 83 articles on the etiology of trauma-related ACS of the forearm [12]. Their systematic review reported that fracture-related ACS was the most frequent (65.4%), followed by cases involving soft tissue injury (30.7%), and, subsequently, cases associated with vascular injuries (3.9%) as far as predisposing risk factors for ACS were concerned. Among fractures contributing to forearm ACS, supracondylar humerus fractures stood out as the primary instigator [11].

The findings further indicate a predilection for ACS incidents associated with the forearm radius, particularly within the distal segment or in combination with concurrent injuries involving neighboring bones [11]. In 2009, Hwang et al. conducted a study on ACS incidences in 1,286 patients presenting with a fracture that disrupted the unstable distal portion of the radius and/or an elbow injury (involving fractures at the upper end of the radius and/or ulna, elbow fracture-dislocation, or fractures of the distal humerus), who were treated at two level I trauma centers within five years [13]. The study concluded that patients presenting with a combination of distal radius fracture and elbow injury were 50 times more likely to develop ACS [13].

Soft tissue damage was reported as the second-most common cause of ACS among patients in the articles reviewed. McQueen and Gaston found that elevated pressure within the compartment arises in conjunction with injuries occurring either in the vicinity or within the compartment itself, resulting in the development of ACS [8]. Stella et al. also reported soft tissue damage as the second-most prevalent predictor and cause of leg ACS among patients [14].

Other studies have reported blunt soft tissue injury as secondary to major or minor injuries of the forearm such as the various types of fractures discussed in this review. Khoshhal et al. reported blunt soft tissue damage as a result of trauma of the forearm, both arms, open and/or radial, ulna, and fractures of the distal humerus [15]. The findings of the studies under this review are in line with Kalyani et al. who studied 80 cases of ACS and reported that 33% of forearm intracompartmental was caused by soft tissue damage in the forearm.

Many complications arise from ACS of the forearm, including tissue and nerve damage, Volkmann’s contracture, gangrene, and amputation. The complications have a serious effect on a patient’s quality of life [16,17]. This review therefore serves to demystify the misconceptions around the risk factors for forearm ACS and the associated side effects. We hope that this review serves to aid trauma surgery decision-making by aiding in the early detection and proper diagnosis of the condition and avoiding the adverse outcomes of ACS. This review is merited by a wide selection of samples involving more than 300,000 patients and cases across numerous countries.

Study limitations

This review was limited by the lack of completeness of information presented in many articles. We recommend a systematic review and meta-analysis of ACS and its associated side effects to determine the most common outcome of ACS in patients whose condition was not diagnosed and addressed early.

## Conclusions

The preeminent role of fractures as the principal catalyst for forearm ACS has been underscored, particularly with a spotlight on ulna and radius shaft fractures. Additionally, the influence of soft tissue trauma in elevating the incidence of forearm ACS has been substantiated. The review’s synthesis emphasizes the significance of meticulous consideration of these risk factors in clinical decision-making, facilitating early detection, accurate diagnosis, and proactive intervention. By acknowledging these factors, healthcare providers can navigate ACS-associated complexities with heightened precision, ultimately improving patient outcomes and enhancing the quality of care.

## Appendices

Preferred Reporting Items for Systematic Reviews and Meta-Analysis checklist

1. Title: Identify the report as a systematic review - “Predictors of Acute Compartment Syndrome in Patients With Forearm Fractures: A Systematic Review.”

2. Abstract: See the PRISMA 2020 for Abstracts checklist. Provides a structured summary, including background, methods, results, and conclusions. Specifies the number of studies reviewed and the total number of participants - Seven articles reviewed involving over 300,000 patients.

3. Rationale: Describe the rationale for the review in the context of existing knowledge - acute compartment syndrome (ACS) is a critical orthopedic emergency, and understanding its predictors in forearm fractures aids early detection and intervention.

4. Objectives: Objective(s) or question(s) the review addresses - Provides an explicit statement of the identifiers and analyzes predictors of ACS in patients with forearm fractures.

5. Eligibility criteria: Specify the inclusion and exclusion criteria for the review and how studies were grouped for the syntheses. Inclusion criteria - Research articles investigating the risk factors associated with the development of ACS in patients who sustained forearm fractures. Exclusion criteria - Studies combining ACS risk factor analysis with other conditions, intervention studies, medical surveys targeting specific patient groups, or disease cohorts such as diabetes. Preference was given to English-language articles. The study design had to involve original quantitative data analysis. Studies were grouped based on the types of interventions, exposures, and outcomes related to ACS in patients with forearm fractures.

6. Information sources: Specify all databases, registers, websites, organizations, reference lists, and other sources searched or consulted to identify studies. Specify the date when each source was last searched or consulted - Databases: PubMed, Google Scholar, NLM, and ScienceDirect. Reference lists of identified articles were also consulted to identify studies.

7. Search strategy: Present the full search strategies for all databases, registers, and websites, including any filters and limits used - The article provides information about the databases used (PubMed, Google Scholar, and ScienceDirect) and mentions the utilization of MeSH terms and keywords, but the full search strategies are not provided.

8. Selection process: Specify the methods used to decide whether a study met the inclusion criteria of the review, including how many reviewers screened each record and each report retrieved, whether they worked independently, and, if applicable, details of automation tools used in the process - Two independent reviewers screened each record and report retrieved. Disputes were resolved through discussion or a third co-investigator’s decision. No mention of automation tools.

9. Data collection process: Specify the methods used to collect data from reports, including how many reviewers collected data from each report, whether they worked independently, any processes for obtaining or confirming data from study investigators, and, if applicable, details of automation tool used in the process - Cochrane Consumers and Communication Review Group Template was used. Two co-investigators and a librarian independently extracted information from eligible articles. Disputes were resolved through discussion or a third co-investigator’s decision.

10. Data items: List and define all outcomes for which data were sought. Specify whether all results that were compatible with each outcome domain in each study were sought (e.g., for all measures, time points, analyses), and, if not, the methods used to decide which results to collect - Primary outcome: ACS in traumatic forearm fracture patients. Secondary outcome: association between ACS and types of forearm fractures.

11. Data items: List and define all other variables for which data were sought (e.g., participant and intervention characteristics, funding sources). Describe any assumptions made about any missing or unclear information - Variables include first author, publication year, origin, evidence level, sample size, average age, sex ratio, injury type, injury mechanism, ACS risk factors, intervention type, and complications.

12. Study risk of bias assessment: Specify the methods used to assess the risk of bias in the included studies, including details of the tools used, how many reviewers assessed each study and whether they worked independently, and, if applicable, details of automation tools used in the process - The Newcastle-Ottawa Scale (NOS) was used by two independent reviewers. No mention of automation tools.

13. Effect measures: Specify for each outcome the effect measure(s) (e.g., risk ratio, mean difference) used in the synthesis or presentation of results - For the primary outcome, the measure of effect is ACS in traumatic forearm fracture patients.

14. Synthesis methods: Describe the processes used to decide which studies were eligible for each synthesis (e.g., tabulating the study intervention characteristics and comparing against the planned groups for each synthesis (item #5)) - The article does not explicitly detail the processes used to decide which studies were eligible for each synthesis, such as tabulating study intervention characteristics and comparing them against the planned groups.

15. Synthesis methods: Describe any methods required to prepare the data for presentation or synthesis, such as handling of missing summary statistics, or data conversions - The article does not provide specific details on methods required to prepare the data for presentation or synthesis, such as handling missing summary statistics or data conversions.

16. Synthesis methods: Describe any methods used to tabulate or visually display results of individual studies and syntheses - The article does not specify the methods used to tabulate or visually display results of individual studies and syntheses.

17. Synthesis methods: Describe any methods used to synthesize results and provide a rationale for the choice(s). If meta-analysis was performed, describe the model(s), method(s) to identify the presence and extent of statistical heterogeneity, and software package(s) used - Comprehensive Meta-Analysis Version 2.2064 was used for data analysis. Effect sizes were synthesized using the number of patients with and without risk factors for ACS. Heterogeneity was assessed using Cochran’s Q statistic.

18. Synthesis methods: Describe any methods used to explore the possible causes of heterogeneity among study results (e.g., subgroup analysis, meta-regression) - The article does not provide specific details on the methods used to explore possible causes of heterogeneity among study results, such as subgroup analysis or meta-regression.

19. Synthesis methods: Describe any sensitivity analyses conducted to assess the robustness of the synthesized results - The article does not explicitly mention sensitivity analyses conducted to asses the robustness of the synthesized results.

20. Reporting bias assessment: Describe any methods used to assess the risk of bias due to missing results in a synthesis (arising from reporting biases) - The article does not provide specific information about the methods used to assess the risk of bias due to missing results in a synthesis arising from reporting biases.

21. Certainty assessment: Describe any methods used to assess certainty or (confidence) in the body of evidence for an outcome - The article does not explicitly mention the methods used to assess certainty or confidence in the body of evidence for outcomes.

22. Study selection: Describe the results of the search and selection process, from the number of records identified in the search to the number of studies included in the review, ideally using a flow diagram - The initial search yielded 1,476 records. After the screening process, 972 records were excluded based on title and abstract evaluation. A further 461 studies were excluded during a thorough assessment, leaving 43 records eligible for full-text review. After a rigorous quality appraisal, 36 studies were excluded, resulting in the inclusion of seven high-quality studies.

23. Study selection: Cite studies that might appear to meet the inclusion criteria but were excluded, and explain why they were excluded - The article does not provide specific details about studies excluded by name or citation. It mentions exclusion based on criteria such as studies published before 1998, non-English-language publications, and studies irrelevant to the focal theme.

24. Study characteristics: Cite each included study and present its characteristics - The included studies involved patients with forearm fractures, and characteristics such as fracture types, soft tissue damage, and other risk factors were considered.

25. Risk of bias in studies: Present assessments of risk of bias for each included study - The NOS was used by two independent reviewers to assess the quality of included articles, resulting in their classification as “High” or “Low” quality.

26. Results of individual studies: For all outcomes, present, for each study: (a) summary statistics for each group (where appropriate) and (b) an effect estimate and its precision (e.g., confidence/credible interval), ideally using structured tables or plots - The article mentions the use of Comprehensive Meta-Analysis Version 2.2064 but does not provide specific results of statistical syntheses, summary estimates, or measures of statistical heterogeneity.

27. Result of synthesis: For each synthesis, briefly summarize the characteristics and risk of bias among contributing studies - The article does not provide a detailed summary of the characteristics and risk of bias among the contributing studies for each synthesis.

28. Result of synthesis: Present results of all statistical syntheses conducted. If a meta-analysis was done, present for each the summary estimate and its precision (e.g., confidence/credible interval) and measures of statistical heterogeneity. If comparing groups, describe the direction of the effect - The article mentions the use of Comprehensive Meta-Analysis Version 2.2064 but does not provide specific results of statistical syntheses, summary estimates, or measures of statistical heterogeneity.

29. Result of synthesis: Present results of all investigations of possible causes of heterogeneity among study results - Information on investigations of possible causes of heterogeneity among study results is not provided.

30. Result of synthesis: Present results of all sensitivity analyses conducted to assess the robustness of the synthesized results - Specific results of sensitivity analyses are not provided.

31. Reporting bias: Present assessments of risk of bias due to missing results (arising from reporting biases) for each synthesis assessed - Information on assessments of risk of bias due to missing results is not provided.

32. Certainty of evidence: Present assessments of certainty (or confidence) in the body of evidence for each outcome assessed - Information on assessments of certainty in the body of evidence is not provided.

33. Discussion: Provide a general interpretation of the results in the context of other evidence - The findings emphasize the crucial role of fractures and soft tissue damage in forearm ACS, contributing valuable insights to trauma surgery.

34. Discussion: Discuss any limitations of the evidence included in the review - The review acknowledges limitations, such as potential bias and incomplete information in some articles.

35. Discussion: Discuss any limitations of the review processes used - External evaluators used the Epidemiological Appraisal Instrument to assess and assign quality scores, addressing potential biases.

36. Discussion: Discuss implications of the results for practice, policy, and future research - The review suggests further research through systematic reviews and meta-analyses to explore ACS outcomes in undiagnosed cases, aiming to improve early detection and diagnosis.

37. Registration and protocol: Provide registration information for the review, including register name and registration number, or state that the review was not registered - The article was registered in the PROSPERO registry with the identification number CRD42023416344.

38. Registration and protocol: Indicate where the review protocol can be accessed, or state that a protocol was not prepared - The study protocol can be accessed through PROSPERO.

39. Registration and protocol: Describe and explain any amendments to the information provided at registration or in the protocol - This systematic review was conducted and reported in line with the Synthesis without meta-analysis (SWiM) in systematic reviews, a subset of the Preferred Reporting Items for Systematic Reviews and Meta-Analysis (PRISMA) reporting guideline.

40. Support: Describe sources of financial or non-financial support for the review, and the role of the funders or sponsors in the review - The article had no financial support or funding.

41. Competing interests: Declare any competing interests of review authors - The article declares no competing interests of the authors.

42. Availability of data, code, and other materials: Report which of the following are publicly available and where they can be found: template data collection forms; data extracted from included studies; data used for all analyses; analytic code; any other materials used in the review - The article does not specify whether template data collection forms, extracted data, analytical code, or any other materials used in the review are publicly available or where they can be found.

The study protocol was also registered in the PROSPERO registry with the identification number CRD42023416344. Detailed information on the study protocol is provided below.

Systematic review protocol

1. Review title: Predictors of Acute Compartment Syndrome in Patients With Forearm Fractures: A Systematic Review.

2. Anticipated or actual start date: 01/04/2023.

3. Funding sources/sponsors: None.

4. Conflicts of interest: None.

5. Review question: What are the most predictable risk factors associated with the development of ACS in patients with forearm fractures?

6. Searches: PubMed, NLM, Google Scholar, and ScienceDirect.

7. URL to search strategy: file:///C:/Users/Randa/Downloads/Document%20(17).pdf.

8. Condition or domain being studied: Compartment syndrome is a condition in which there is increased pressure within an enclosed fascial compartment space that decreases perfusion and compromises the function of the space contents. Forearm compartment syndrome (FCS) is considered a less common condition compared to the upper extremity but can potentially lead to serious complications.

9. Participants/population: Inclusion criteria: All articles evaluating ACS in patients with forearm fractures. All study designs. All articles with a clear interventional method. All articles were conducted worldwide. All ages. Exclusion criteria: Articles evaluating any type of compartment syndrome without fractures. Articles that examined ACS in any location other than the forearm. Articles with a small unrepresentative sample size. Articles written in any language other than English. Articles conducted on non-human subjects.

10. Intervention(s): Long-arm cast, functional brace, surgical fixation, closed or open reduction and internal fixation, open exposure(s), Reduction with plate and screw fixation, dynamic compression plate, limited contact dynamic compression plate, bridging plate, intramedullary nailing, and hybrid fixation.

11. Comparator(s)/control: All patients with forearm fracture and predictors without ACS.

12. Context: Development of ACS.

13. Main outcome(s): The primary objective is to identify the risk factors associated with the development of ACS in patients with forearm fractures.

14. Measures of effect: Outcome is defined as ACS in traumatic forearm fracture patients.

15. Additional outcome(s): The secondary objective is to evaluate whether there is any association between ACS and type of forearm fracture.

16. Data extraction (selection and coding): Cochrane Consumers and Communication Review Group Template will be used to extract data from the included articles. Two co-investigators and a librarian will be assigned to extract independently the information from all the articles that meet the study eligibility criteria and any dispute will be resolved by a discussion or a third co-investigator’s decision.

17. Information that will be elicited includes the articles’ first author, publication year, origin, evidence level, sample size, average age, sex ratio, injury type, injury mechanism, ACS risk factors, intervention type, and complications. A third co-investigator will then be assigned to review the accuracy and integrity of the extracted information.

18. Risk of bias (quality) assessment: The NOS will be used by two independent reviewers to assess the quality of the included articles and their risk of bias by awarding points to each study based on three main domains: cohort selection, group comparability, and study outcome.

19. Strategy for data synthesis: In the case and control group, effect sizes will be synthesized using the number of patients with and without risk factors for ACS. Furthermore, if a particular risk factor is found to be reported by more than one article, odds ratio or relative hazards will be used and then the data will be converted to OR. Heterogeneity will be assessed by computing Cochran’s Q statistic of included articles. For low sensitivity concerns, statistical significance will be considered with a p-value of less than 0.10. Publication bias will be assessed using the Begg-Mazumdar test and funnel plot.

20. Analysis of subgroups or subsets: If the existing data are sufficient, risk factors will be assessed as subgroups

21. Type and method of review: Systematic review.

22. Health area of the review: Surgery.

23. Language: English.

24. Country: Ireland and Saudi Arabia.

25. Keywords: Medical subject headings (MeSH) terms, including “forearm fracture,” “acute compartment syndrome,” and “compartment syndrome,” were paired with epidemiological terms like “survey” and “systematic review.” Boolean operators such as “AND” and “OR” were incorporated into the search strategy to combine these keywords effectively.
